# Multiple intra-abdominal splenosis with imaging correlative findings: A case report and review of literature

**DOI:** 10.1016/j.radcr.2024.06.084

**Published:** 2024-07-26

**Authors:** Ashok Chapagain, Gopal K. Yadav, Sandeep Bhandari, Karun Devkota, Bijay Adhikari, Ashish Singh, Raksha Bhattarai

**Affiliations:** Department of Radiodiagnosis and Imaging, BP Koirala Institute of Health Sciences, Dharan, Nepal

**Keywords:** Abdomen, Computed tomography, Splenosis

## Abstract

We present a case of a 31-year-old male who presented to the emergency department with a history of abdominal pain localized to the lower abdomen. The patient had undergone splenectomy 2 years ago for splenic injury following a road traffic accident. Computerized tomography showed multiple well-defined, homogeneously enhancing soft tissue density nodules of varying sizes distributed throughout the abdomen and pelvis. A diagnosis of splenosis was made based on imaging findings and history. Abdominal splenosis is an uncommon entity of which radiologists need to be aware, and this case serves to shed further light on this condition.

## Introduction

Splenosis, an autoimplantation of focal splenic tissue deposits in various body compartments, often occurs following trauma or surgery [1]. Despite both splenunculus and splenosis being ectopic splenic tissues, their pathophysiologies differ. Splenunculus results from incomplete fusion of mesenchymal buds during embryogenesis, while splenosis stems from post-traumatic or postsurgical implantation. It's identified in about a quarter of patients who undergo splenectomy due to trauma [2,3]. Typically asymptomatic, splenosis is incidentally detected during imaging for unrelated reasons [4]. However, it has been diagnosed in cases where it manifested as persistent regional chest pain [5,6], hemoptysis [7] or occult gastrointestinal bleeding [8], and bowel obstruction [9]. While histopathological diagnosis remains the gold standard, imaging supported by the patient's history of splenectomy or prior spleen trauma is deemed acceptable. Further investigations are unnecessary unless there's a potential confusion with other clinical conditions [10].

In this instance, we present a case involving a 31-year-old male who recently underwent splenectomy due to traumatic splenic injury. The patient exhibited symptomatic abdominal splenosis distributed across the hypochondriac, lumbar, and pelvic regions.

## Case presentation

A 31-year-old male, who had undergone splenectomy 2 years ago due to traumatic splenic injury, presented to the emergency department (ED) with abdominal pain persisting for 1 week. The pain, diffuse across the abdomen with greater intensity in the lower region, had progressively worsened and did not respond to over-the-counter oral analgesics. He denied experiencing vomiting, fever, recent travel, or any changes in stool color. The patient's medical history revealed a prior splenectomy following abdominal injuries sustained in a motor vehicle accident.

During examination, the patient appeared mildly distressed. His pulse, blood pressure, respiratory rate, and temperature were recorded at 108 bpm, 136/82 mm Hg, 20 cycles/min, and 98.2°F respectively. Abdominal assessment revealed softness, nondistention, and mild tenderness in the hypogastrium, without signs of venous engorgement or abnormal appearance. Lung auscultation revealed clear sounds, and his heart exhibited normal sounds.

**Fig. 1:**
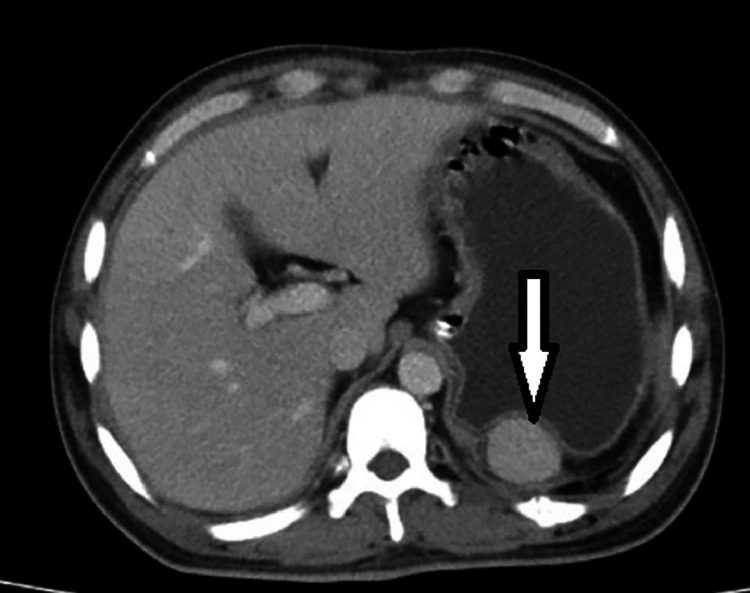
Axial contrast enhanced CT image showing absence of spleen and splenic implant in left hypochondrium.

**Fig. 2:**
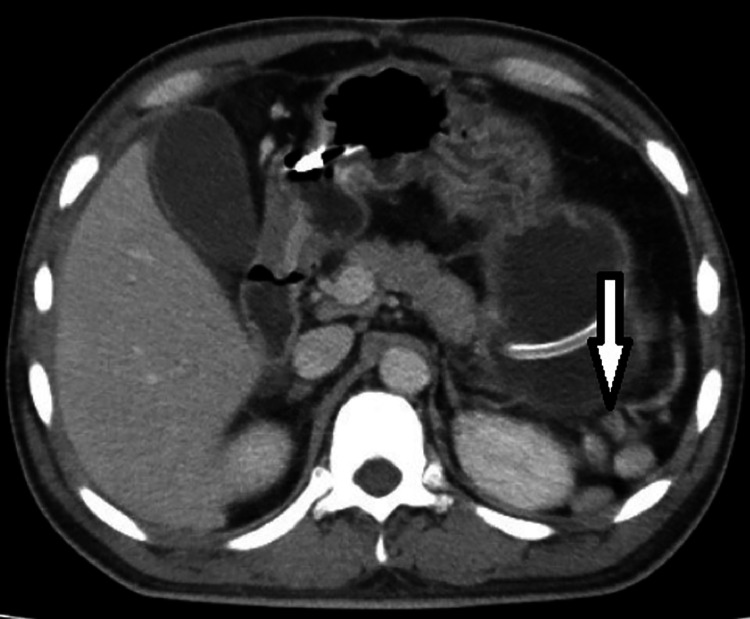
Axial contrast enhanced CT image showing splenic implants in left lumbar region.

**Fig. 3:**
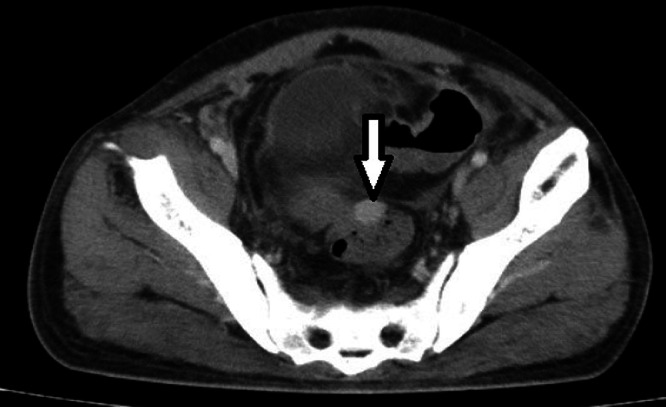
Axial contrast enhanced CT image showing splenic implant in pelvis abutting sigmoid colon.

**Fig. 4:**
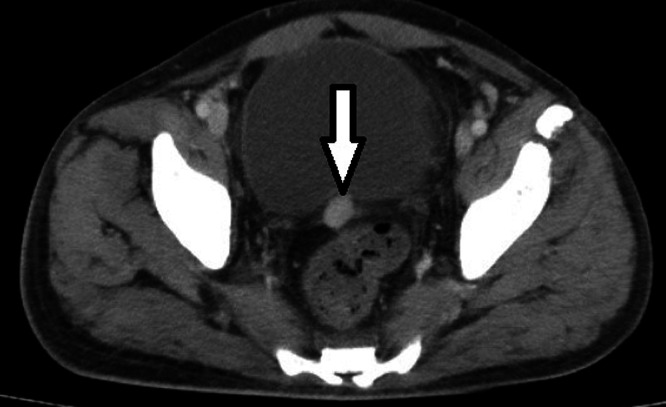
Axial contrast enhanced CT image showing splenic tissue implant in pelvis abutting posterior wall of urinary bladder.

Due to the abdominal pain, an abdominal ultrasound was conducted, which did not detect any calculi in the kidney, ureter, or bladder and yielded normal results. Subsequently, a contrast-enhanced CT scan of the abdomen and pelvis was recommended. The CT scan revealed the absence of the spleen in its usual location and the presence of multiple well-defined, homogeneously enhancing soft tissue nodules of varying sizes distributed throughout the abdomen and pelvis. These were identified as splenic implants, with the largest measuring about 3.2 × 2.5 cm^2^. Eight splenic implants were visible on the imaging: 1 in the left hypochondrium anteriorly, abutting the greater curvature of the stomach; 5 in the left lumbar region adjacent to and lateral to the upper pole of the left kidney; and 2 in the pelvis—one adjacent to the sigmoid colon and the other against the posterior wall of the urinary bladder (Fig. 1, Fig. 2, Fig. 3, Fig. 4). The diaphragmatic wall remained intact. Based on the history of splenectomy and the presence of multiple well-defined enhancing nodules, a diagnosis of splenosis was established.

The patient received treatment in the ED, including intramuscular analgesics (intravenous ketorolac 30 mg stat) and intravenous antacids (intravenous pantoprazole 40 mg stat), and was observed for several hours before discharge. He was prescribed oral analgesics (400 mg ibuprofen tablets thrice daily for 3 days) with instructions to follow up after a week. During a 6-month follow-up, the patient did not report any recurrence of abdominal pain.

## Discussion

Splenosis commonly occurs in areas such as the peritoneum, omentum, and mesentery, but reports also indicate splenic implantation in unexpected locations like the pericardium, subcutaneous tissue, and even within the brain. The implanted splenic tissue possesses the ability to derive blood supply from surrounding tissues and vessels, functioning akin to a normal spleen [11].

While typically asymptomatic, these splenic deposits might occasionally lead to recurrent abdominal pain or, in rarer cases, small bowel obstruction due to adhesive bands formed by splenic implants. Other infrequent presentations include gastrointestinal bleeding, infarction or hematoma formation within nodules, abdominal masses, hydronephrosis due to ureteral compression, or recurrence of hematologic diseases previously treated with splenectomy [12].

Heterotopic implantation of splenic tissues following abdominal trauma or splenectomy remains an underdiagnosed condition. The actual incidence of this benign condition remains unknown as it's usually incidentally diagnosed during imaging or surgical procedures. These implants can be solitary or multiple, occurring throughout the peritoneal cavity or chest [13]. Maintaining a high index of suspicion in patients with relevant medical history is crucial for diagnosis. Physicians should consider this rare condition based on a comprehensive medical history, thorough physical examination, and the absence of typical changes in blood smear postsplenectomy, such as Howell-Jolly bodies or reticulocytosis [14,15].

Imaging techniques like abdominal ultrasound, contrast-enhanced CT scan, and magnetic resonance imaging (MRI) offer limited diagnostic value in managing abdominal splenosis. On contrast-enhanced CT scans, they exhibit density and enhancing characteristics similar to the expected density of the spleen in patients with splenectomy [16]. In MRI, the signal characteristics in splenosis resemble those of a normal spleen, appearing hypointense on T1-weighted imaging, hyperintense on T2-weighted imaging, and demonstrating heterogeneous enhancement on contrast-induced MRI. However, the splenic deposits may appear hypointense on T2-weighted imaging in the presence of iron deposition [17].

Due to the nonspecific imaging features of splenosis, it can be mistaken for various conditions such as metastatic disease, abdominal lymphoma, hemangiomatosis, peritoneal mesothelioma, multifocal endometriosis, primary renal or hepatic malignancies, gliomatosis peritonei, granulomatous peritonitis (as seen in tuberculosis or histoplasmosis), tumor rupture, or reactive adenopathy [11].

Nuclear scintigraphy remains the preferred diagnostic modality for splenosis. While the Tc-99m sulfur colloid test can localize the reticuloendothelial system, scintigraphy using Technetium-99m heat-damaged erythrocytes (RBC) or Indium 111-labeled platelets is currently considered more sensitive and specific for splenic uptake, making them the preferred diagnostic tools [18].

Recent case reports have highlighted the use of ferumoxides-enhanced MRI as an innovative tool for diagnosing splenosis. Ferumoxides, superparamagnetic iron oxides, are eliminated from circulation by the reticuloendothelial system, including splenic tissue [19].

Regarding management, asymptomatic splenosis usually requires conservative treatment, while severe symptomatic cases may warrant surgical intervention [20,21]. However, prospective studies are needed to understand the consequences of splenosis and guide appropriate management.

## Data availability statement

Not applicable.

## Ethical approval

Not applicable.

## Provenance and peer review

Not commissioned, externally peer-reviewed.

## Role of generative AI

None.

## Author contributions

AC was involved in the conception of the report. AC, GKY, SB, KD, BA, AS and RB were literature review, initial manuscript preparation, manuscript critique and review, and the final manuscript preparation. All authors have read and approved the final manuscript.

## Patient consent

Written informed consent was obtained from the patient for publication of this case report and accompanying images. A copy of the written consent is available for review by the Editor-in-Chief of this journal on request.
